# Sinapic Acid and Its Derivatives as Medicine in Oxidative Stress-Induced Diseases and Aging

**DOI:** 10.1155/2016/3571614

**Published:** 2015-11-10

**Authors:** Chunye Chen

**Affiliations:** Key Laboratory of Biorheological Science and Technology, Ministry of Education, Bioengineering College, Chongqing University, Chongqing 400030, China

## Abstract

Sinapic acid (3,5-dimethoxy-4-hydroxycinnamic acid) is an orally bioavailable phytochemical, extensively found in spices, citrus and berry fruits, vegetables, cereals, and oilseed crops and is known to exhibit antioxidant, anti-inflammatory, anticancer, antimutagenic, antiglycemic, neuroprotective, and antibacterial activities. The literature reveals that sinapic acid is a bioactive phenolic acid and has the potential to attenuate various chemically induced toxicities. This minireview is an effort to summarize the available literature about pharmacokinetic, therapeutic, and protective potential of this versatile molecule in health related areas.

## 1. Introduction

As a result of metabolic processes, there is continuous production of reactive oxygen species (ROS), such as hydroxyl radicals [1], in human body. Various biological functions like antimicrobial activity depend on ROS [2]. In normal physiological state, ROS production in body is balanced by scavengers “antioxidants.” This equilibrium is disturbed in pathological conditions owing to overproduction of ROS, but comparatively low concentration of endogenous antioxidants in body. It results in the reaction between ROS and intra- and extracellular species leading to emergence of oxidative stress which causes various ailments like aging, cancer, and necrosis [3]. To tackle the oxidative stress, it is needed to restore balance between ROS and antioxidants by administering exogenous antioxidants, for example, hydroxycinnamic acids.

Hydroxycinnamic acids belong to the class of phenolic acids with bioactive carboxylic acids; the class mainly includes caffeic acid, ferulic acid, and sinapic acid [4, 5]. According to literature, these compounds are capable of donating their phenoxyl hydrogen atom for neutralization of free radical species leading to production of corresponding phenoxyl radicals. These radicals are weekly reactive due to delocalization of unpaired electrons. Resultantly, the inhibition of dangerous radicals is useful for human health owing to antiaging potential of these phenolic acids [6, 7].

**Figure 1:**
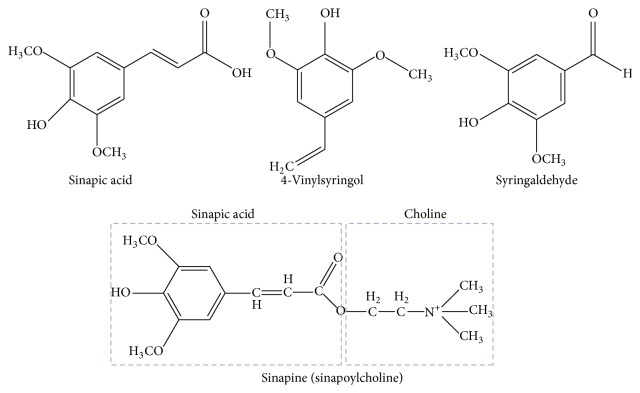
The structural formulas of sinapic acid and its derivatives (syringaldehyde, sinapine, and 4-vinylsyringol).

Sinapic acid exists in both free and ester form; some esters are sinapoyl esters, sinapine (sinapoylcholine), and sinapoyl malate [8, 9]. Sinapic acid is a phytochemical found in various edible plants such as spices, citrus and berry fruits, vegetables [10–12], cereals, and oilseed crops [13, 14]. Sinapic acid has been tested and reported against various pathological conditions such as infections [15], oxidative stress [16], inflammation [17, 18], cancer [19], diabetes [20], neurodegeneration [21], and anxiety [22]. Some derivatives of sinapic acid, such as sinapine, 4-vinylsyringol, and syringaldehyde, have also been studied for acetylcholinesterase inhibition [23, 24], antimutagenicity [25], and antioxidant activity [26], respectively. 4-Vinylsyringol, a decarboxylated sinapic acid, is also termed as canolol. The term “canolol” was coined by Wakamatsu et al. due to its source, canola oil [25]. The structural formulas of sinapic acid and its derivatives are shown in Figure 1 [23, 27, 28]. The literature search does not show any extensive research on the biological features of sinapic acid and its derivatives. Those studies have been summarized in this brief review article so that the scientific community may pay more attention to the biological aspects of sinapic acid and its derivatives.

## 2. Pharmacokinetics of Sinapic Acid

Fruit and vegetable consumption can potentially decrease the risk of degenerative diseases which mainly attributed to the phenolics present in them. Pharmacokinetic study helps to understand the role of these phenolics in human body. Serum albumin has been reported to be responsible for the transport of sinapic acid in blood due to its ability to bind with serum albumin through hydrophobic interaction and hydrogen-bonding [29, 30]. Maximum plasma-sinapic acid level has been described as 40 nM with a bioavailability of 3% of the total phenolics present in the nonprocessed cereal meal [31, 32]. Moreover, the small intestine was reported as the best place for absorption of orally administered sinapic acid through active Na^+^ gradient-driven transport [33]. Plasma-sinapic acid level has also been quantified (1.5 *μ*g/mL) after intake of cranberry juice in human by using GC-MS [34]. However, metabolism of sinapic acid takes place in the epithelium of the small intestine [35]; urine analysis, after sinapic acid ingestion in rats, showed the presence of sinapic acid, 3-hydroxy-5-methoxyphenylpropionic acid, methyl sinapate-sulfate, methyl sinapate-glucuronide, dihydrosinapic acid, 3-hydroxy-5-methoxycinnamic acid, and their acid-labile conjugates [35] and these are generated by the esterase activity of the intestinal microflora [32, 36]. Nature of these metabolites also indicates the possible metabolism of free and ester form of sinapic acid through phase I and II reactions in human small intestinal epithelium [37].

## 3. Antioxidant Activity

Reactive oxygen species (ROS) are continuously generated and are used in normal physiologically based activities [38]. Simultaneously, they are captured by different scavengers, known as antioxidants, to maintain their equilibrium in human body [39]. However, the overproduction of ROS destroys this equilibrium resulting in oxidative stress which is responsible for various pathological conditions, such as cancer, neurodegenerative disorders, and aging [40–42]. Polyphenols consist of four major classes of phytochemicals, that is, phenolic acids, flavonoids, stilbenes, and lignans [43], and behave as antioxidants, useful as anticancer, antiaging, and antimicrobial agents and scavengers of ROS produced in the body [44, 45]. Presence of methoxy- and hydroxyl-groups in the structure of polyphenols also improves their antioxidant ability [45, 46]. Sinapic acid belongs to this family of phenolics with remarkable antioxidant potential. Various modes of antioxidant activity of sinapic acid have been documented in the literature as described below.

### 3.1. DPPH^•^ Scavenging Potential

Sinapic acid is also known to show free radical scavenging ability against paramagnetic stable radical of* 2,2*-diphenyl-*1*-picrylhydrazyl (DPPH^•^). According to the literature, the DPPH^•^ inhibition by 0.02 mM, 0.5 mM, and 0.3 mM of sinapic acid is 33.2% [8], 88.4% [47], and 50% [48], respectively. Moreover, 8-8′-bis-lactone-dimer of sinapic acid also shows DPPH^•^ scavenging activity but at concentrations higher than 200 *μ*M [48].

Additionally, sinapic acid derivatives like sinapoyl glycosides are also reported for DPPH^•^ scavenging activity [49, 50]. However, these studies report the higher DPPH^•^ radical scavenging activity of sinapic acid as compared to its glycosides including sinapoyl glucose, sinapine, and 6-O-sinapoyl sucrose except methyl 2-O-sinapoyl-*α*-D-glucose and methyl 6-O-sinapoyl-*α*-D-glucose which showed a little higher activity than that of sinapic acid.

Synergism in DPPH^•^ scavenging activity of sinapic acid is also observed; however, comparatively higher antioxidant potential of rapeseed meal and oil extracts has been reported which contains 4-vinylsyringol (87% w/w) and sinapine (13% w/w) along with sinapic acid, in comparison with pure sinapic acid alone [54]. In addition, the DPPH^•^ scavenging activity of sinapic acid is also compared with its derivatives, for example, 4-vinylsyringol; however, DPPH^•^ scavenging activity of sinapic acid (90.8%) was described to be higher than that of 4-vinylsyringol (78.7%) at a concentration of 1 mg/mL [54–56]. Moreover, another derivative syringaldehyde is also reported to show strong DPPH^•^ scavenging activity [26, 57].

### 3.2. O_2_^∙−^ Scavenging Potential

Superoxide anion radical (O_2_^∙−^) can suppress [4]-containing dehydratases and oxidize some compounds including leukoflavins, tetrahydropterins, and catecholamines. However, O_2_^∙−^ scavenging activity of sinapic acid has been found similar to that of 4-vinylsyringol (decarboxylated product of sinapic acid), which shows that the decarboxylation of sinapic acid does not modify its O_2_^∙−^ scavenging activity [56]. Moreover, an excellent O_2_^∙−^ scavenging activity of sinapic acid (IC50 = 17.98 mM) has been reported in comparison with Trolox used as an antioxidant (IC50 = 7.24 mM) [17]. In another study, O_2_^∙−^ inhibition was presented 35.52% by using 0.05 mM of sinapic acid [58], in both enzymatic (IC50 = 70.7 *μ*M) and nonenzymatic (IC50 = 979.2 *μ*M) O_2_^∙−^ generating systems. Moreover, the O_2_^∙−^ scavenging activity of sinapoyl glycosides is also reported; however, this study reports the lower O_2_^∙−^ radical scavenging activity of sinapic acid (IC50 = 90 mM) as compared to its glycoside, 6-O-sinapoyl sucrose (IC50 = 65 mM) [59].

### 3.3. ^•^OH Scavenging Potential

Highly reactive hydroxyl radicals (^•^OH) have potential to damage their surroundings in living system [60, 61]. Sinapic acid has been reported as a good scavenger for ^*∙*^OH with an IC50 = 3.80 mM where ascorbic acid was used as standard showing IC50 = 5.56 mM [62]. Moreover, three ester derivatives of sinapic acid, methyl sinapate, *β*-D-(3,4-disinapoyl)fructofuranosyl-*α*-D-(6-sinapoyl)glucopyranoside, and 1,2-disinapoyl-*β*-D-glucopyranoside, have also shown comparable ^•^OH scavenging activity [63].

### 3.4. Scavenging Potential against Other Free Radicals

Sinapic acid has been known for hydroperoxyl radical (^•^OOH) scavenging activity [64, 65]; however, 4-vinylsyringol, a derivative of sinapic acid, scavenges the ^•^OOH more quickly than sinapic acid [65, 66].

Sinapic acid also possesses better ClO^−^ scavenging potential as compared to other hydroxycinnamic acids, that is, ferulic acid, chlorogenic acid, and* p*-coumaric acid. Sinapic acid has also been reported to be efficient nitric oxide radical (^•^NO) scavenger compared to the reference compound, that is, 2-(4-carboxyphenyl)-4,4,5,5-tetramethylimidazoline-1-oxyl-3-oxide potassium salt [17].

Peroxynitrite (ONOO^−^) can potentially initiate apoptosis [65]. Sinapic acid has been described to perform better ONOO^−^ scavenging activity by inhibiting 3-nitrotyrosine formation in protein (bovine serum albumin) through an electron donation mechanism as compared to standard antioxidants, that is, ascorbic acid, penicillamine, and tocopherol [65]; however, sinapic acid scavenging activity against ONOO^−^ further increases in the presence of 25 mM Na_2_CO_3_, which contribute CO_2_ for simulation of physiological environment [17, 67]. In addition, 4-vinylsyringol can also scavenge ONOO^−^ [11].

### 3.5. Suppression of Lipid Peroxidation

Lipid peroxidation generates lipid hydroperoxides, which act as a source of lipid peroxyl (LOO^•^) and lipid alkoxyl (LO^•^) radicals [68]. In a comparative study, sinapic acid was compared with *α*-tocopherol and ferulic acid on the formation of hydroperoxides, and results showed that sinapic acid acts more efficiently to suppress the hydroperoxide formation by preventing the lipid oxidation in bulk methyl linoleate [16, 69]. Moreover, in another comparative study, the antioxidant potential of sinapic acid was compared with other antioxidants, that is, Trolox and butylated hydroxyanisole [70, 71]. Sinapic acid at a concentration of 500 *μ*mol/kg has been found comparable in lipid peroxidation inhibition against Trolox and butylated hydroxyanisole; the results are even better than *α*-tocopherol. Similarly, the concentration-dependent inhibition of hydroperoxide formation by sinapic acid and sinapine was observed in purified rapeseed oil stored at 40°C in darkness; however, sinapine was found to be noneffective on hydroperoxide synthesis inhibition alone [49].

In another study, the prooxidant behavior of sinapine in rapeseed oil was reported and is attributed to its low solubility in oil [71]. An inverse relationship has been explained between the antioxidant property of sinapic acid and the concentration of tocopherols because sinapic acid may lose its function due to reaction with tocopherol radicals whose concentration got increased in elevated tocopherol level. Furthermore, an increased amount of sinapic acid is reported to produce less quantity of propanal (secondary oxidation product) at low tocopherol concentration and larger quantity at high levels. Concisely, sinapic acid can potentially play a role in the stability of oils containing small quantities of endogenous tocopherols [71].

Lipid peroxidation can be affected by sinapic acid derivatives. In a comparative study, 15% more antioxidant activity of 4-vinylsyringol has been observed against sinapic acid in a nonpolar system; however, a diminished activity of 4-vinylsyringol is reported in polar environment [60]. In another study, 4-vinylsyringol was found to be a more potent RCOO^•^ scavenger than vitamin C and *α*-tocopherol [25]. Moreover, a promising peroxyl radical scavenging activity of syringaldehyde has been reported in crocin method, involving a competition between antioxidant and crocin to bind with the peroxyl radical; a similar antioxidant activity of syringaldehyde has been published in bulk oil and lecithin liposome [29]. Similarly, in another study, liposome (lipid membrane model) was used to assess lipid peroxidation capacity of sinapic acid and was found to be an excellent protective agent for the membrane, especially when added at the liposome synthesis stage [72]. Furthermore, linoleic acid-based lipidic model was used and the diferential scanning calorimetric analysis of sinapic acid, its alkyl esters (methyl, ethyl, propyl, and butyl sinapates), and reference antioxidant (Trolox) was conducted to compare their peroxyl radical scavenging activity. The results revealed that the test substances had reducing abilities comparable to that of reference compound suggesting sinapic acid and its alkyl esters as promising antioxidants [73].

*(1 ) Inhibition of Oxidation of Low-Density Lipoprotein (LDL)*. Low-density lipoprotein (LDL) oxidation has been found responsible for atherosclerosis development [74]. In a comparative study, sinapic acid showed higher (28%) antioxidant activity than 4-vinylsyringol (7.5%), in a LDL model system at a concentration of 10 *μ*M [54]. Moreover, peroxyl radicals produced through Cu^+2^-mediated oxidation of human LDL has been studied* in vitro*, and in terms of Trolox equivalent (TE) the following order has been observed with decreasing lipid peroxidation inhibition capacity: sinapic acid > caffeic acid > ferulic acid [75]. Additionally, concentration-dependent inhibition of LDL oxidation by sinapic acid has also been reported which can be attributed to its chelating power with Cu^+2^ [76–78]. Similarly, Cu^+2^-mediated peroxidation of human LDL and peroxyl radical can attack on erythrocyte membranes resulting in AAPH- (2,2′-azobis(2-amidinopropane) dihydrochloride-) induced hemolysis; however, ethyl sinapate at a concentration of 10 *μ*M was found to act more effectively (76%) and suppressed the LDL oxidation than sinapic acid (59%). Moreover, in terms of IC50 values, for 50% AAPH-induced hemolysis inhibition capacity, the studied hydroxycinnamates can be configured in the following decreasing order: sinapic acid (IC50 = 4.5 *μ*M) > ethyl sinapate (IC50 = 5.0 *μ*M) > caffeic acid (IC50 = 7.2 *μ*M) > ferulic acid (IC50 = 6.8) [79].

### 3.6. Anti-Inflammatory and Anticarcinogenic Properties

Nitric oxide synthase, tumor necrosis factor-*α* (TNF-*α*), cyclooxygenase-2, and interleukin-1*β* are proinflammatory mediators and their expression by ROS and activated nuclear factor-kappa B (NF-*κ*B) in macrophages cause inflammation [19]. Inflammation produced by incorrect regulation of NF-*κ*B disturbs immunity and can produce autoimmune diseases, that is, cancer [80]; however, a suppressive action of sinapic acid on NF-*κ*B has been reported in the literature [18, 81]. Moreover, sinapic acid has been described to have time-dependent and dose-dependent suppressive effect on colon and breast cancer cells (human breast cancer T47D cell line) and this inhibitory action is attributed to its antiproliferative feature [19, 82]. Furthermore, proinflammatory mediators are reported to be suppressed by 4-vinylsyringol [83]. In another study, sinapic acid and its alkyl esters were evaluated for anti-inflammatory activity in carrageenan-induced rat paw oedema model and an excellent anti-inflammatory activity of isopentyl sinapate was reported in comparison to other esters [84].

The ROS are generated due to* Helicobacter pylori* (*H. pylori*) infection, which attack and damage macromolecules, including DNA, fats, and proteins. Therefore, damaged DNA produces 8-hydroxy-2′-deoxyguanosine (8-OHdG); however, its level can be reduced by 4-vinylsyringol treatment [85]. In Mongolian gerbils infected with* H. pylori*, oral administration of 4-vinylsyringol (0.1% in the diet) has been described to efficiently suppress the gastric malignancy [83]. In an* in vivo* study, the protective effect of canolol against inflammatory bowel disease and colitis associated carcinogenesis via inhibition of inflammatory cytokines and oxidation stress was observed [86]. Same effect of canolol has also been reported in human retinal pigment epithelium (ARPE-19) cell line through an extracellular signal regulated kinase-mediated antioxidative pathway [87]. Additionally, canolol has also been found capable of inhibiting bacterial (*H. pylori*) mutation by protecting DNA damage from ONOO^−^, a highly oxidative chemical [88]. Peroxynitrite radicals (ONOO^−^) can cause DNA cleavage resulting in mutation [80]. Sinapic acid and 4-vinylsyringol have been studied for their antimutagenic characteristics and it was reported that both hydroxycinnamic acids have potential and dose-dependent antimutagenicity character, possibly through ONOO^−^ scavenging action [25].

### 3.7. Anxiolytic Property

Elevated plus-maze (EPM) and hole-board test are generally used for anxiolytic studies in mice [89]. These tests were employed to study the behavior of sinapic acid and it was found that it increases the time spent in open arms significantly and also increases percentage entry in open arms [22]. Moreover, due to no side effects of sinapic acid even after its prolonged use and its selective anxiolytic features in comparison to existing anxiolytic agents [90, 91] a targeted research is required to use sinapic acid preferably in anxiety conditions.

### 3.8. Neuroprotective Property

Few studies are available in the literature, which elaborate the neuroprotective function of sinapic acid and its derivatives. Sinapine, a derivative of sinapic acid, during* in vitro *studies has been found to have dose-dependent acetylcholine (ACh) esterase inhibitory activity; moreover, sinapine and ACh both contain quaternary nitrogen to bind reversibly to specific region on AChE in a competitive mode [23, 24]. Furthermore, activity of sinapine is more effective in the cerebral homogenate than in blood serum of rats with IC50 values of 3.66 *μ*M and 22.1 *μ*M, respectively [23].

### 3.9. Antimicrobial Activity

Emergence of drug resistance in microbes is a fast growing issue in health sciences. Drugs available in market are constantly facing the problem of drug resistance, and therefore new drug molecules are required to counter this threat [92–94]. In* in vivo *studies, conducted on various Gram-positive and Gram-negative bacteria, 97–99% eradiation of various microorganisms was observed indicating significant antibacterial potential of sinapic acid [95].  Table 1 carries minimum inhibitory concentrations (MIC) of sinapic acid against various bacterial strains observed during* in vitro* studies. In another study, sinapic acid was reported to have the potential to selectively kill the pathogenic bacteria leaving beneficial lactic acid bacteria alive that can resist and metabolize the sinapic acid [14]. Moreover, syringaldehyde has been described for its antifungal potential against* Candida guilliermondii* [96].

### 3.10. Antihyperglycemic Activity

Antihyperglycemic activity of sinapic acid was reported using induced-hyperglycemic* in vivo *model [97, 98] by intraperitoneal administration (45 mg/kg body weight) of streptozocin (STZ, a compound which destroys the insulin secreting pancreatic-cells). Subsequently, both normal and hyperglycemic rats were studied for certain biochemical markers (blood urea, serum creatinine, uric acid, total protein, albumin, and A/G ratio) and hepato- and nephron-histopathology; however, altered values of the studied biochemical markers and pathological features came to normal state after treating the rats with sinapic acid (15 mg/kg and 30 mg/kg) for 35 days; therefore, sinapic acid may have dose-dependent hepato- and nephron-protective effects in STZ-induced-hyperglycemic rats. In addition, sinapic acid can be further studied for applications in diabetic states.

### 3.11. Antilipidemic Activity

One of the causative agents of cardiovascular diseases, such as myocardial infarction, is abnormal lipid profile of a subject [99]. In this context, a study involving antilipidemic activity of sinapic acid has been proposed by Roy and Prince [100]. They administered isoproterenol (100 mg/kg body weight) to rats for inducing myocardial infarction, and then the myocardial infarcted rats (rats with raised levels of cardiac troponin-T, cholesterol, triglycerides, and free fatty acids in serum and higher ST-segments in electrocardiogram) were studied to evaluate the shielding effects of sinapic acid [100, 101]. Recently, during* in vivo* studies performed on rats, an orally administered sinapic acid dose (12 mg/kg body weight) showed shielding effects on hypertrophy of heart, abnormal lipid levels, and electrocardiogram; furthermore, pre- and cotreatment with sinapic acid standardized the levels of myocardial infarction parameters which further elaborate antioxidant potential as well as antilipidemic activity of sinapic acid. Moreover, lysosomal dysfunction in isoproterenol-induced myocardial infarcted rats can also be cured by sinapic acid [102, 103]. These evidences elaborate the antilipidemic activity of sinapic acid.

### 3.12. Toxicities and Sinapic Acid

#### 3.12.1. Isoproterenol-Induced Myocardial Infarction

Isoproterenol (ISO), a synthetic catecholamine, can cause the lysosomal lipid peroxidation [103] followed by the production of various lysosomal enzymes, such as lysosomal hydrolases [104], which produce myocardial infarction (MI) [105]. The ISO-mediated lysosomal dysfunction in rats suffering from MI can be overcome by oral administration of sinapic acid in rats at a concentration of 12 mg/kg body weight. This effect is evident from the changes in lysosomal lipid peroxidation, serum lysosomal enzymes, heart homogenate, lysosomal fraction, and myocardial infarct size calculated before and after simultaneous intake of sinapic acid. The treatment with sinapic acid notably suppressed the ISO-provoked release of lysosomal enzyme activity, normalized all the biochemical parameters, and diminished myocardial infarct size [102]. The membrane stabilizing features and free radical scavenging potential of sinapic acid can be the possible mode for the above-mentioned activities [104]. Thus, sinapic acid may be employed as a protective agent in MI [102].

#### 3.12.2. Kainic Acid-Induced Hippocampal Neuronal Damage

Neuron depolarization and extreme calcium influx by kainic acid (KA, a nonselective agonist of AMPA and kainate receptors) generate the free radicals, activate the nitric oxide synthase (NOS), and initiate the mitochondrial dysfunctioning [106, 107]; it results in glutamatergic activation- and oxidative stress-mediated inflammation and neurodegeneration [108, 109]. Sinapic acid has been evaluated due to its GABA receptor agonistic feature and free radical scavenging potential, during* in vivo *study in rats, for new glutamate receptors blockers and radical scavengers for neuroprotection. An oral administration of sinapic acid at a concentration of 10 mg/kg body weight was reported to efficiently treat the KA-induced brain damage. However, the neuroprotective effect of sinapic acid was attributed to its radical scavenging potential and anticonvulsive activity through GABA receptor activation [110, 111].

#### 3.12.3. Amyloid *β* (A*β*) _1–42_ Protein-Induced Alzheimer's Disease

Neuroprotective effect has been studied in mouse suffering from Alzheimer's disease, a neurological disease involving cognitive impairment [112, 113], and was induced in mouse by amyloid *β* (A*β*)_1–42_ protein injected into the hippocampus. Simultaneously, after injecting A*β*_1−42_ protein; an oral administration of sinapic acid was started with a dose of 10 mg/kg body weight per day. A*β*_1−42_ protein-induced effects were reported to be abolished by the use of sinapic acid, including elevated expression of iNOS, glial cells, and nitrotyrosine. Similarly, in rats suffering from cognitive impairment induced by scopolamine, sinapic acid shows better results [21]. Moreover, promising neuroprotective effects were reported in rodents, where sinapic acid suppressed potassium cyanide-induced hypoxia and scopolamine-induced memory impairment [114].

#### 3.12.4. Carbon Tetrachloride and Dimethylnitrosamine-Induced Acute Hepatic Injury

Carbon tetrachloride (CCl_4_) can produce the proinflammatory mediators causing an acute hepatic inflammation and its associated pathologies [115]. Sinapic acid has been described for its potential to revert the CCl_4_ intoxication of liver by oral administration of 10 or 20 mg/kg body weight in rats. Moreover, the sinapic acid treatment notably suppressed the CCl_4_-provoked release of proinflammatory mediators by scavenging the free radicals [116]. Sinapic acid has the potential to be used as a remedial approach for inhibiting hepatic inflammation [117–119]. Moreover, sinapic acid has also effectively treated dimethylnitrosamine-induced hepatotoxicity [120].

#### 3.12.5. Corticosterone-Induced Toxicity

Corticosterone administration in broiler chickens can produce oxidative stress, which retards the animal growth. Corticosterone-induced toxicity can be countered by the use of 4-vinylsyringol to preserve the tissue *α*-tocopherol level and to reduce the lipid peroxidation in the animal. Therefore, 4-vinylsyringol can also be added to broiler chicken feed to exert effective antioxidant effect [121].

#### 3.12.6. *tert*-Butyl Hydroxide-Induced Toxicity

Antioxidant potential of 4-vinylsyringol against* t*-BH- (*tert*-butyl hydroxide-) mediated production of ROS, which induce the human retinal epithelial cell death, has been studied and compared to a standard antioxidant,* N*-acetyl cysteine; however, it has been reported that 4-vinylsyringol at a concentration of 200 *μ*M exerts more protective effect than the reference compound [122].

#### 3.12.7. Arsenic-Induced Toxicity

Arsenic can cause pathological conditions like cancer and diabetes on long-term exposure [123, 124] by disturbing various enzymatic reactions in liver resulting in generation of ROS (superoxide, peroxyl radicals, and hydrogen peroxide) which produce hepatotoxicity. During* in vivo *study, arsenic-induced toxicity can be shielded by the use of sinapic acid and is mainly attributed to its metal-chelating potential [62]. Therefore, sinapic acid administration can help in avoiding arsenic-induced toxicity [125].

### 3.13. Toxicity Study of Sinapic Acid

The toxicity profile of sinapic acid has been reported to be considerably low in broiler chickens; no effect on the serum activity of creatine kinase and lactate dehydrogenase has been reported and observed. Therefore, it is not harmful to various body organs of the animal [126].

## 4. Conclusion

Sinapic acid and its derivatives, particularly 4-vinylsyringol, are interesting natural compounds that has potential to express various health benefits, that is, antioxidant, anti-inflammatory, anticancer, antimutagenic, antiglycemic, neuroprotective, and antibacterial activities. Moreover, further extensive and targeted studies are required to explain relationship between the plasma concentrations of sinapic acid, in therapeutic dose, and the therapeutic outcomes.

## Figures and Tables

**Table 1:** Minimum inhibitory concentrations (MIC) of sinapic acid against bacteria strains.

Number	Reference	Bacterial strain	Minimum inhibitory concentrations (MIC)
		*Bacillus subtilis *	0.45 g/L
1	Barber et al., 2000 [51]	*E. coli *	0.89 g/L
		*Pseudomonas syringae *	1.79 g/L

		*E. coli *	0.49 g/L
2	Tesaki et al., 1998 [52]	*Salmonella enteritidis *	0.45 g/L
		*Staphylococcus aureus *	0.43 g/L

3	Engels et al., 2012 [14]	*Bacillus subtilis *	0.3 g/L
		*E. coli *	0.7 g/L
		*Staphylococcus aureus *	0.3 g/L
		*Listeria innocua *	0.3 g/L
		*Listeria monocytogenes *	0.2 g/L
		*Pseudomonas fluorescens *	0.6 g/L

4	Johnson et al., 2008 [53]	*Salmonella enteric *	Not mentioned
